# Bridging HIV prevention and sexual reproductive health services in the context of multi-method PrEP: a qualitative study exploring provider perceptions in South Africa

**DOI:** 10.3389/frph.2026.1746212

**Published:** 2026-02-27

**Authors:** Faith Mary Musvipwa, Siphokazi Dada, Fatima Abegail Cholo, Melanie Pleaner, Mosa Julia Letsielo, Sean Arries, Alison Kutywayo, Catherine Elizabeth Martin, Saiqa Mullick

**Affiliations:** Wits RHI, University of the Witwatersrand, Johannesburg, South Africa

**Keywords:** contraception, healthcare provider, pre-exposure prophylaxis, service integration, sexual reproductive health

## Abstract

**Introduction:**

The introduction of new pre-exposure prophylaxis (PrEP) methods presents an opportunity to expand choice and better integrate HIV prevention and broader sexual and reproductive health (SRH) services. Integrating HIV prevention services, including PrEP, and SRH services presents an opportunity to improve healthcare access and outcomes. However, implementation within real-world healthcare settings requires an understanding of both enablers and barriers from the perspective of healthcare providers (HCPs).

**Methods:**

A qualitative descriptive study was conducted using in-depth interviews (IDIs) to describe the integration of PrEP and SRH services, drawing on the perspectives of HCPs. Thirty-four HCPs were purposively sampled and interviewed between October and November 2024 across four South African locations: eThekwini, Gqeberha, Mthatha, and Tshwane. Audio recordings were transcribed, translated, and analysed using inductive thematic analysis with NVIVO 14 software.

**Results:**

Key benefits of service integration expressed by HCPs included increased continuity of care with the same HCP, convenience, and a higher uptake of SRH services, such as contraception and sexually transmitted infection (STI) screening and management. Facilitators included integrated education and awareness, flexibility in alignment of appointment schedules, and positive staff attitude. Challenges and barriers included misalignment between injectable PrEP and injectable contraceptive visit schedules and infrastructural limitations within some public health facilities.

**Conclusions:**

Successful integration requires adaptable systems, responsive scheduling, functional infrastructure, and positive staff attitude. To improve uptake and continuity of SRH services, health systems should be flexible and provide holistic services that align with client needs.

## Introduction

Despite progress in HIV prevention, critical gaps remain in meeting the intersecting sexual and reproductive health (SRH) needs of different populations, particularly adolescent girls and young women (AGYW) (1). Globally, HIV remains a significant public health challenge, with an estimated 39.9 million people living with HIV at the end of 2023, 65% of whom are in the African region (2). In 2023, approximately 1.3 million people acquired HIV, significantly exceeding the global target of fewer than 370,000 new infections by 2025 (3). While young women account for a disproportionate share of new HIV infections in the region, unmet need for contraception also remains high, with nearly one in six young women (15–19 years) in sub-Saharan Africa reporting an unmet need for family planning (4). The introduction of new pre-exposure prophylaxis (PrEP) methods presents an opportunity to expand choice and better integrate HIV prevention and broader SRH services (5, 6). In a context marked not only by the evolving HIV epidemic and the introduction of new prevention methods, but also by critical declines in international and donor funding, health systems face an urgent imperative to strengthen their capacity to deliver integrated, differentiated, and equitable interventions (3). Integration has the potential to increase efficiencies in service delivery and resource utilization (7, 8). Integration can be structural, built into the design of health systems, or functional, developed in response to specific client or programmatic needs (9–12). Gaps in access to both contraception and HIV prevention remain as significant challenges for young women globally, particularly in sub-Saharan Africa (13). These intersecting SRH needs underscore the urgency of integrated approaches that can enhance choice and effective use of available methods (13). Meeting SRH needs for AGYW requires comprehensive and person-centred service delivery models (14). Integrated HIV prevention services create opportunities for contraception, sexually transmitted infection (STI) management, and reducing HIV vulnerability (15–17). For AGYW, such integration is especially impactful as it addresses intersecting challenges and helps overcome the fragmentation and burdens of navigating health systems. Healthcare providers (HCPs) play a central role in the implementation and success of HIV prevention and SRH integration (18). As noted in our previous work, integrated services provide important benefits, in particular, the use of PrEP serves as an entry point for enhancing access to contraception and related SRH services (19, 20). This paper builds on this work and aims to describe the perspectives of HCPs and the facilitators and barriers to service integration for PrEP and SRH, in the context of new PrEP method (long-acting injectable cabotegravir [CAB-LA] and dapivirine vaginal ring [DVR] introduction in South Africa. While much of the existing literature has focused on oral PrEP delivery, there are limited data on service integration in the context of multi-method PrEP. To our knowledge, this is the first study to report on healthcare provider perspectives on integration in a setting where multiple PrEP methods are being introduced. Understanding provider views is critical, as their attitudes, capacity, and experiences directly influence how integration is implemented, the quality of care delivered, and the extent to which clients can access and benefit from a range of HIV prevention options.

## Methods

### Study setting

This study was conducted as part of the work of Project PrEP, an implementation science study funded by Unitaid that aims to inform HIV prevention delivery models and expand choice among AGYW in South Africa through the provision of accessible, differentiated, and integrated HIV prevention and SRH services. The project approach has been described previously for oral PrEP introduction (21). The project is carried out in four diverse areas of South Africa: eThekwini (KwaZulu-Natal), Gqeberha and Mthatha (Eastern Cape), and Tshwane (Gauteng), where activities in each location are undertaken in two fixed public healthcare clinics alongside a roving mobile clinic. Since August 2023, three of the four sites (Gqeberha, Mthatha, and Tshwane) have provided the DVR alongside oral PrEP, with CAB-LA introduced in April 2024. Participants in the ongoing parent cohort are offered a choice of PrEP methods in line with national guidelines (22, 23). Cohort participants are HIV-negative, ≥15 years, and interested in HIV prevention services, including those who are PrEP naïve, as well as prior or current PrEP users. At these sites, different cadres of HCPs are responsible for delivering various components of PrEP and SRH services according to national guidelines (24–26). Their roles include PrEP education, choice counselling, PrEP initiation, follow-up monitoring, and the integration of PrEP services within broader SRH services. Site-based clinical staff consists of professional nurses, managers, and a roving clinical mentor. Non-clinical staff include lay counsellors and client navigators. Professional nurses provide clinical services such as PrEP choice counselling, PrEP provision, STI syndromic management and contraception. Lay counsellors are responsible for HIV testing and counselling, and client navigators provide health education, generate demand for services, and guide clients through the health facility.

### Study design

This study employed a phenomenological descriptive qualitative research design to describe the perspectives of HCPs on the facilitators and barriers to service integration for PrEP and SRH, in the context of new PrEP methods introduction in South Africa. The study adhered to Consolidated Criteria for Reporting Qualitative Research (COREQ) (Supplementary Appendix 1) guidelines to ensure methodological rigor (26).

### Sampling, recruitment, and study population

HCPs were recruited through a purposive sampling approach to identify cadres actively delivering PrEP services to ensure representation of both clinical staff (professional nurses) and non-clinical staff (counsellors and client navigators). Eligible participants were required to be at least 18 years old, actively providing SRH and PrEP services at the study sites, and willing to participate in an audio-recorded interview. Thirty-six providers meeting these criteria were invited: thirty-four participated, with two declining (one unavailable, one non-response). Recruitment was done telephonically and through email by a senior researcher who is not involved in the day-to-day activities at the sites. Informed consent was obtained before participation, and demographic information was collected to provide a contextual background for the analysis.

### Data collection

Data were collected through single individual in-depth interviews (IDIs) between October and November 2024 with thirty-four HCPs aged 25–63 years. Among those who participated fifteen were clinical staff (professional nurses) and nineteen were non-clinical staff (counsellors and client navigators). Interviews were held in private spaces within the healthcare facilities or nearby community-based organisations (CBOs) to ensure confidentiality, while three were conducted telephonically. To help limit social desirability bias, interviews were conducted by three trained female qualitative researchers (FAC, FMM, and SD) not involved with the participants’ day-to-day work activities, and participants were assured of confidentiality and the non-evaluative nature of the study. Interviews were conducted using a semi-structured guide developed by the research team to explore experiences, perceptions, training needs, and delivery models for different PrEP products; however, the focus of this paper is on service integration. The interview guide was not piloted as it was adapted from previous similar research conducted among HCPs and eliminated the need for pretesting. All data were anonymised, stored securely on access controlled server on Microsoft teams, and accessible only to the research team. Interviews were conducted in English, isiZulu, Setswana, or isiXhosa, depending on the participants’ language preference, and lasted on average 60 min. All interviews were audio-recorded with participant consent. To compensate for their time participants received a reimbursement of R300.

### Data analysis

All interviews were transcribed verbatim in the original language, translated into English where necessary, and reviewed for accuracy before analysis. Translations were reviewed by bilingual team members to verify accuracy and preserve meaning. Any discrepancies were discussed and resolved before analysis to ensure cross-language validity. A thematic analysis was conducted following Braun and Clarke's six-phase steps (27, 43) to systematically identify and interpret key themes. We captured diverse perspectives, which resulted in breadth rather than reaching data saturation on a single aspect. Sample sufficiency was determined by including participants with varied professional roles, backgrounds, and service delivery experiences to capture a broad range of perspectives relevant to the study aim. This breadth-oriented approach aligns with the phenomenological design, which prioritizes variation in lived experiences rather than saturation within narrowly defined categories. This approach ensured a comprehensive understanding of HCPs’ perspectives on the integration of PrEP and SRH services. NVivo 14 software was used for data management and coding. Three researchers (FAC, FMM, and SD) independently each conducted open coding on two transcripts, compared their coding for consistency, and collaboratively developed a preliminary coding framework. This framework was further refined and finalized through discussions with the broader study team, which included four researchers (FAC, FMM, SD, and AK) and two HIV technical specialists (CEM and MP). The finalized framework was then systematically applied to the remaining transcripts to ensure consistency and depth in thematic analysis.

### Researcher characteristics and reflexivity

Analysis was primarily conducted by FAC, FMM, and SD in discussion with the broader research team. FAC is a female researcher with a Master's degree in Research Psychology and experience in qualitative research. FMM is a female researcher with a PhD in sociology and experience in qualitative research. SD is a female researcher with a PhD in Public Health and experience in qualitative research. All three analysts have prior experience in SRH and HIV-related qualitative research, which informed familiarity with the study context. To address positionality and potential bias, the analysts engaged in reflexive practices throughout analysis, including discussing emerging interpretations with the broader research team. These discussions were used to critically reflect on how researchers’ backgrounds and assumptions might influence interpretation and to ensure findings remained grounded in participants’ accounts.

### Ethical considerations

The study received ethical approval from the Human Research Ethics Committee at the University of the Witwatersrand (220604) and the World Health Organization (WHO) Ethics Research Committee (0003784). Written informed consent was obtained from all participants for both their participation and the audio recording of the interviews.

## Results

Table 1 presents a summary of the participant's demographic information. The results are presented across three key areas of service integration: benefits; facilitators; and challenges or barriers. Reported benefits of service integration included increased continuity of care with the same provider, convenience, and improved uptake of SRH and HIV prevention services, including PrEP, contraception, and STI screening. Key facilitators of integration were education and awareness efforts, flexibility in alignment of appointment schedules, and positive staff attitude. Despite these benefits and facilitators, challenges were also identified, such as misalignment between CAB-LA and injectable contraceptive schedules and infrastructural limitations within some health facilities.

**Table 1:** Socio-demographic characteristics of study participants.

Socio-demographic characteristics	*N* (=34)	%
Age group		
25–34 years	21	61,8
35–44 years	9	26,5
45 + years	4	11,8
Biological sex		
Male	4	11,8
Female	30	88,2
Geographical area		
Tshwane	9	26,5
Mthatha	10	29,4
eThekwini	7	20,6
Gqeberha	8	23,5
Staff category		
Clinical staff	15	44,12
Non-clinical staff	19	55,88
Months in current job		
0–12 months	4	11,8
13–24 months	15	44,1
25 + months	15	44,1

### Benefits of service integration

i) Increased continuity of care with the same healthcare provider

Continuity of care, where clients see the same provider, was mentioned as a key benefit of integrated services. HCPs reported that when clients consistently engage with the same provider for multiple services, it strengthens trust, reduces the need to explain themselves every time, and fosters a more supportive on-going provider-client relationship. This theme reflects relational consistency over time.

“… you screen the client that is on CAB-LA for STI, you check pregnancy, every time the client comes. You check if the client has an STI, and then you track accordingly if the client has it or not. So, you screen every time the client comes for pregnancy and STIs.” (Clinical staff, Mthatha)

“…you build like a better rapport and the relationship with the client, then clients would come back to say, I'm looking for that nurse and I only want to see that nurse because they know, like they don't have to re-explain themselves, like what was the issue last month.” (Clinical staff, Tshwane)

HCPs mentioned that when clients return to a familiar provider, they feel more comfortable and will return for services.

“…they get familiar with one person, if they know that this is the nurse that I'm comfortable with, then they know that they can always come back.” (Non-clinical staff, Tshwane)

ii) Convenience

A key benefit of service integration was the creation of a “one-stop shop” where clients could access contraception, STI screening and management, and HIV prevention services in a single visit under the same roof. HCPs mentioned that this reduced the need for clients to have multiple appointments. HCPs reported that clients valued receiving multiple services in one room. This one-stop-shop model was seen as highly convenient, saving clients time and effort by eliminating the need to queue at multiple service points hence reflecting structural and logistical efficiency of service delivery. HCPs emphasized that integrated service delivery is especially beneficial for clients with limited time. Clients were reported to value the ability to plan their day around predictable and streamlined service provision, which enhances their overall healthcare experience and encourages continued engagement. Without service integration, HCPs noted, clients might be deterred from seeking care due to the time burden of visiting multiple service areas.

“… some of our clients are students and they come here after school, especially around half past two, three o'clock. And because of the integration of services that we have, they [clients] know that even though I go to the facility at three o'clock, I would always find it and then I'm going to leave at a certain time. I'm not gonna spend the whole day.” (Non-clinical staff, Tshwane)

“Mostly, there is this thing of one-stop shop. It helps that a patient must not sit in this room for contraception and go to that room for PrEP and go to that room for STI. It is good for them to do everything in one consultation to eliminate time.” (Non-clinical staff, Mthatha)

iii) Increased uptake of SRH services

Integrating HIV prevention and SRH services encourages clients to engage with a broader range of preventive healthcare services. HCPs reported that clients who initially seek treatment for STIs or access contraception are introduced to other prevention interventions, such as PrEP, leading to increased uptake. Education during consultations helps shift perceptions, encouraging clients to take proactive steps in their sexual health. This also leads to an interest and desire to try biomedical HIV prevention products. Additionally, peer influence was also reported to play a role, with clients referring friends after receiving services and information. HCPs leveraged integration to offer comprehensive care, addressing other health concerns during the same visit.

“…you find out that the adolescent girls and young women are only coming for contraception, they do not know anything about PrEP. So, when you talk to them about PrEP methods, you will see that they are interested in taking PrEP.” (Non-clinical staff, Gqeberha)

“… they [clients] come for STI and they're not even aware that they're at the risk of HIV. But then, when they get here, because we are integrating services, they get to learn about PrEP, and then someone would now realize I’m at risk of getting HIV.” (Non-clinical staff, Mthatha)

### Facilitators for service integration

i) Education and awareness

An important facilitator of the successful integration of new PrEP methods and SRH services was comprehensive, integrated education and awareness. HCPs emphasized the need to inform clients about available SRH services, including contraception and STI screening, HIV testing, and HIV prevention options, in an integrated manner. HCPs highlighted that young people often avoid coming to the clinic; therefore, when they do visit, it is crucial to provide all services holistically to prevent missed opportunities.

“…So, we need to understand that they [young people] don't want to come to the clinic, so we need to try, when they are here, to do everything that they need. So, we check what they came here for, you provide all the services that we have, all the products for PrEP. Now you also check the contraceptive. So, we need to look at everything holistically. We don't look at one thing because they won't come back.” (Clinical staff, Mthatha)

“Because when the client comes to the clinic, they [nurses] provide them with information on these products and SRH services. So, a client gets a lot. The client goes home having received contraceptives and knowing her HIV status.” (Non-clinical staff, Mthatha)

“Mostly it’s the education… They might have come in not for PrEP, but for something else. And while sitting in the waiting room, they have a client navigator speaking to them about PrEP. Then they come to your PrEP line. That’s how they end up there.” (Clinical staff, eThekwini)

ii) Flexibility in alignment of appointment dates

To reduce the number of times clients must come to the clinic, HCPs need to be flexible and problem-solve ways PrEP and contraception visits can be aligned. The schedules for contraception and PrEP method were not always well aligned. A key strategy was aligning PrEP and contraceptive appointments, particularly for those using the injectable contraception methods, to enable clients to receive both services in a single visit. This not only improved convenience but also reduced transport costs and time burdens for clients. This client-centred problem-solving approach facilitated integrated service delivery, enhanced access to PrEP, and supported continuity of care.

“..So, the client was saying, how about I miss coming to my contraception and wait until my PrEP date? …she wanted her clinic dates to be aligned [so that] when she comes for contraception, she can also take CAB-LA.” (Clinical staff, Gqeberha)

“…now we can align the dates for those who are on contraceptives. So then, at least we can align their dates. When they come for contraception, they're also coming for the PrEP.” (Clinical staff, Tshwane)

HCPs reported that flexibility and problem-solving were helpful, for example, where oral contraceptives were used as a bridge to align the client's PrEP method and injectable contraceptive appointments. HCPs noted that alignment of oral contraceptives with DVR was easier than for CAB-LA, whereas schedule alignment posed greater challenges with CAB-LA, making this a key barrier to integration.

“Oral contraceptives, so that next month when she comes for the second injection, [CAB-LA] we offer both the injectable contraceptive and PrEP. So that we can align the dates. Oral contraceptives are aligned better, and with DVR, they don't have a problem. You can align it with any contraceptive.” (Clinical staff, Mthatha)

For clients initiating PrEP for the first time, careful scheduling was required to ensure alignment with contraception appointments. HCPs reported that they adjusted follow-up appointments to ensure that new PrEP users could gradually transition to aligned dates by adjusting the scripting of the oral PrEP or contraceptive methods. Additionally, HCPs mentioned that misaligned visit schedules also influenced clients’ decision-making around contraception, with some women switching or choosing specific contraceptive methods to better align with their PrEP appointments and reduce the need for frequent clinic visits.

“…choice counselling has influenced the client to choose their contraception accordingly. …remember when the client comes, we align the appointment days. …with CAB-LA because it’s a two-monthly injection, first one month first, then two monthly. So, it also has contributed to clients changing their contraceptive methods just to make sure that, they are aligned because they don't want to come to the facility now and then. (Clinical staff, Tshwane)

“…you need to align all of that because you would have a new PrEP initiation, never been on contraception come to you for PrEP services. You initiate them on PrEP, offer contraception, and they want to go on the oral contraceptive. …You review them in a month’s time and give them one month of PrEP and one month of contraception… So, that is how you align the two. If it’s an injectable method, the client is coming on a two monthly injectable method, you do an initiation, you give them the two monthly injectable method. In a month’s time you recall them for PrEP for month one follow-up. But then again, you give them another month so that they come back just to align it with their two monthly methods [contraception] so that moving forward they only get two monthly PrEP and two monthly injectable [contraception] method so that you align them to ensure that the two appointments are being done in a one stop shop manner.” (Clinical staff, eThekwini)

HCPs described proactively working with clients to align visit schedules, sometimes even recommending adjustments to contraception methods to better align with PrEP follow-ups.

“…we tell them take this type of contraception so that next time when you come the dates will match. So, we integrate contraception mostly because most of them come for contraception than PrEP.” (Clinical staff, Tshwane)

iii) Positive staff attitude

HCPs noted that the provision of integrated services must be grounded in quality of care, with staff attitude playing a central role in facilitating interpersonal interaction quality. A positive, respectful, and non-judgmental approach was reported as essential for creating a welcoming environment that supports integration. One HCP reported that simple gestures such as smiling, speaking politely, and avoiding harsh tones contributed to a positive clinic experience and encouraged clients to return for follow-up visits.

“…if the client has been treated the right way at the clinic, they will be happy. Then the client will come back. I also think a positive attitude is important to the client. Because if the client did not get a positive attitude, she would leave and never come back… Even when you talk to them [clients], you mustn’t shout; they don’t want that. Always smile when you talk to them, and they’ll come back.” (Non-clinical staff, Mthatha)

### Challenges and barriers of service integration

i) Aligning three-monthly injectable contraceptives and CAB-LA

HCPs reported that a major challenge in integrating CAB-LA with the client's current contraception method is the misalignment of visit schedules. Since CAB-LA follows an initial one-month injection followed by two monthly injections, it does not align with the three-monthly schedule of injectable contraceptives like depot medroxyprogesterone acetate (DMPA). This results in additional clinic visits for clients, which can be inconvenient, particularly for those with financial constraints, busy school/work schedules or transportation difficulties. HCPs mentioned that they attempted to align schedules by discussing with clients the option to switch to contraceptive methods that align with CAB-LA's dosing schedule. However, HCPs reported that many clients preferred their current contraceptive method due to personal experiences, such as side effects with alternative options. HCPs reported that some clients expressed frustration over needing multiple clinic visits, while others were willing to adjust. Despite the challenge, HCPs focused on clear communication to help clients understand the scheduling implications and potential alternatives.

“…make them understand that maybe some dates are not going to be the same, and then we talk to the client and inform the client that we would love to align their visit dates. And now that is starting the CAB-LA, because they have to take the CAB-LA for one month, firstly, and then two monthly, if the client is on two months injectable [contraception]. We would suggest that the client take one-month oral PrEP” (Clinical staff, Mthatha)

“…sometimes you find out that integration is not going to work based on what the client wants. Because when the client is on DMPA that injectable is three months injectable but if the client is also on CAB-LA, it cannot integrate. We cannot integrate CAB-LA and DMPA, because there will always be that one month that overlaps you, see? So, it depends on which product the client is on because sometimes we try to change the client’s minds to say, for you to be able to integrate this, you maybe have to change from using this one to this one..” (Clinical staff, Mthatha)

“…if the client comes and the client is using maybe the two-month contraceptives, and they want CAB-LA, remember the CAB-LA, you start with one month, and then she’s here for two months injection. So, the dates are not going to align. So that was a challenge for me to convince the client to switch the contraceptive methods..” (Clinical staff, Tshwane)

ii) Healthcare facility infrastructure constraints

Integration needs a supportive health system. Constraints due to infrastructural problems were reported to create barriers to both the quality of care and efforts to integrate services. HCPs explained that in many facilities, pregnancy testing is conducted at designated service points within the facility rather than being integrated into all service areas. Another key issue is the lack of infrastructure within certain clinics, such as running water or functioning toilets, which prevent on-site urine pregnancy testing. Clients are sometimes required to go to other areas of the clinic for urine testing or return home if a pregnancy test cannot be done on-site. This fragments the service, often leading to missed follow-up visits and delays in accessing PrEP and contraception. Mobile services provide a more streamlined approach, offering pregnancy testing within a single setting.

“…in this facility, we struggle a lot there are times when there’s no water and we cannot do pregnancy test, for instance. We cannot do urine testing… So, if we could maybe have a working station that’s gonna have toilets it would help us a lot because when the toilets in the facility are not working, we'll be able to do pregnancy test because sometimes when a client comes in for PrEP, maybe it’s their follow up date, but they are behind with contraception we have to send them where they have [water] or to go home.” (Non-clinical staff, Gqeberha)

“With the mobile it’s much easier because everything happens in the mobile..They know that everything is just in one place. And it’s much quicker that side. With the facility, some of the things we are assisted by the DOH. So, it means we work hand in hand with them. Certain things can delay. Let’s say the likes of urine testing, they need to go to do it that side with the mobile, you have the toilet inside with the facility, you need to travel a certain distance for you to go there…” (Non-clinical staff, Tshwane)

## Discussion

This study describes HCPs’ perspectives on integrating new PrEP methods and SRH services, shedding light on both the operational and relational elements that shape successful integration in real-world settings. Three key domains emerged: the benefits of service integration, facilitators that enable it, and the challenges and barriers that must be addressed. HCPs highlighted how integration improves continuity of care by enabling clients to see the same provider across multiple services, which builds trust, reduces the need for repeated explanations, and improves client comfort, particularly for sensitive issues such as HIV prevention and contraception. PrEP integration was described as a critical component of responsive SRH services. HCPs emphasized that offering PrEP alongside contraception, HIV testing, and STI management allows clients to consider prevention holistically rather than as separate decisions. They noted that counselling on multiple PrEP and contraception methods could be complex, as clients’ choices for one method sometimes influenced the other. At these service points injectables, oral contraceptives, and implants are offered, and uptake of injectable contraceptive methods is high making alignment of CAB-LA and injectable visits of relevance. This service integration not only reduces missed opportunities for initiation but also fosters continuity. Such integration reduces repetition of explanations from the client, aligns appointment schedules, and promotes more efficient use of clinic resources. These findings align with existing literature, which supports that integrated models strengthen client engagement and reduce service fragmentation (28–30). This holistic approach enhances patient trust and retention in healthcare, ultimately leading to better health outcomes (17, 28, 29, 31). The convenience of a “one-stop shop” model also emerged as a major benefit, especially for young people and those with limited time or resources. HCPs perceived that clients valued receiving PrEP, contraception, and STI services during a single visit, as this reduced travel costs and waiting times. This study highlighted reduced costs for clients, which may also translate into broader system-level savings, particularly relevant amid recent reductions in global health funding. These insights are especially pertinent for the upcoming scale-up of six-monthly injectable PrEP (lenacapavir), underscoring the importance of flexible, equitable service models that can accommodate differing product schedules and client preferences. Echoing our findings, other studies suggest that this strategy not only reduces the number of visits required but also helps clients better manage their time and transportation costs (32–34). Importantly, integration was perceived to improve the uptake of multiple SRH services, as clients coming for contraception or STI treatment were introduced to PrEP through routine consultations. This process reflects the “education-interest-try-adopt” pathway described in Briedenhann's EITA model, where client education serves as the essential entry point to engagement with biomedical prevention (35). Similarly, findings from another study revealed that there is a need for continued education and improved accessibility of both contraception and PrEP (36). Our study indicates that key facilitators include comprehensive, integrated education and awareness; flexible alignment of appointments, particularly for injectable PrEP and contraceptive users; and positive, respectful provider attitudes. HCPs described how even small gestures like smiling, speaking politely, and listening to clients had a powerful impact on clinic engagement and return visits, underscoring that effective integration is as much about interpersonal relationships as it is about systems. Consistent with our findings, another study indicated that staff attitude is an influential factor in the quality of service offered in a healthcare facility (13). HCPs also demonstrated willingness to be flexible in aligning visit schedules to suit client needs, reflecting a high degree of service-level flexibility that supported integrated delivery. Similarly, other studies revealed that aligning contraception visits with PrEP visits facilitated integration by optimizing service efficiency and reducing missed opportunities (37, 38). However, integration was not without challenges. Misaligned dosing schedules between CAB-LA (every two months) and injectable contraceptives (either two-monthly or three-monthly) created logistical burdens, often requiring additional clinic visits or difficult decisions about switching contraception methods. HCPs typically tried to align visits for CAB-LA with a two-monthly injectable contraceptive. Many HCPs reported clients preferring to have their visits aligned. Infrastructural issues, particularly related to pregnancy testing, were noted as a barrier to seamless service integration. These challenges reflect broader systemic constraints in the South African public health system, including resource limitations, aging facility designs, and inconsistent availability of basic utilities, which hinder implementation of integrated service models. Previous studies corroborate our findings, asserting that health facility infrastructure has an impact on healthcare services (35, 39). These operational challenges highlight gaps in current service models and underscore the potential value of Multipurpose Prevention Technologies (MPTs) (45), which simultaneously prevent HIV, other STIs, and unintended pregnancy, as well as other innovative products designed to better align prevention needs with integrated service delivery (40–42). Similarly, the integration of longer-acting injectable PrEP (e.g., 6 monthly lenacapavir) may alleviate the need for more complex alignment schedules between PrEP and contraception.

### Limitations

Our findings may not be generalizable to different settings, as we only described the views of HCPs in settings where integration was implemented. We acknowledge the potential for social desirability bias in this study, as data were collected through IDIs with healthcare providers working at the project sites. Given their role within the healthcare system, participants may have provided responses that aligned with institutional expectations or perceived best practices. While our main project focuses on AGYW, integration approaches should consider the perspectives of key populations to determine if similarly integrated models may address their specific preferences and needs.

## Conclusions

The findings presented in this paper offer novel insights into the integration of PrEP and SRH services, particularly in the context of introducing multiple PrEP methods and as longer-acting PrEP methods are introduced. Successful integration of HIV prevention and SRH services requires a holistic, client-centred approach supported by flexible systems, positive provider attitudes, and functional infrastructure. Education and awareness remain key to introducing new biomedical interventions, while flexibility in appointment scheduling enables alignment of product visits and continuity of care. Health systems should plan for the complexity of integrating products with differing clinical requirements and ensure services can be provided efficiently in a single visit.

## Data Availability

The raw data supporting the conclusions of this article will be made available by the authors, without undue reservation.
