# Supplementary material for: Bridging HIV prevention and sexual reproductive health services in the context of multi-method PrEP: a qualitative study exploring provider perceptions in South Africa
Source: Front Reprod Health. 2026 Feb 27;8:1746212. doi: 10.3389/frph.2026.1746212 (PMC12982184; doi:10.3389/frph.2026.1746212)
Supplement: Supplementary file 1 [file Table1.docx]

**APPENDIX 1 - COREQ checklist**

The Consolidated Criteria for Reporting Qualitative Studies (COREQ): 32-item checklist

| Item Guide questions/description Notes | Item Guide questions/description Notes | Item Guide questions/description Notes |
| --- | --- | --- |
| Domain 1: Research team and reflexivity | | |
| The research team  The research team was multidisciplinary, bringing together expertise from various fields. It included professionals with backgrounds in nursing, public health, medicine, psychology and social sciences. Team members held advanced degrees such as PhDs, master’s degrees, and medical degree, including MBChB with specializations in tropical medicine and HIV management. This diverse expertise ensured a well-rounded approach to the study. | | |
|  | | |
| Personal  Characteristics | | |
| 1. Interviewer/facilitator | Which author/s conducted the interview or focus group? | FAC, FMM and SD conducted the interviews. |
| 2. Credentials | What were the researcher’s  credentials? E.g. PhD, MD | FAC: MA  FMM: PhD  SD: PhD |
| 3. Occupation | What was their occupation at the time of the study? | FAC: Researcher  FMM: Researcher  SD: Senior Researcher |
| 4. Gender | Was the researcher male or female? | All interviewers were female (FAC), (FMM) and (SD.) |
| 5. Experience and  training | What experience or training did the researcher(s) have? | FAC, FMM and SD have experience in qualitative research methods including  conducting in-depth interviews. |
| Relationship with participants | | |
| 6. Relationship  established | Was a relationship established prior to study commencement? | Participants were informed about the purpose of the interviews before data collection, which was covered in the ICF. Additionally, before the interview, the interviewer reiterated the purpose of the interview to the participants. |
| 7. Participant  knowledge of the  interviewer | What did the participants know about the researcher? e.g. personal goals, reasons for doing the research | Participants knew the researchers as they are staff for the project however researchers were not involved in their daily work activities. |
| 8. Interviewer  characteristics | What characteristics were reported about the inter viewer/facilitator?  e.g. Bias, assumptions, reasons and interests in the research topic. | To help limit social desirability bias, interviews were conducted by three trained female qualitative researchers (FAC, FMM, and SD) not involved with the participants’ day-to-day work activities, and participants were assured of confidentiality and the non-evaluative nature of the study. |
| Domain 2: Study design | | |
| Theoretical framework | | |
| 9. Methodological  orientation and  Theory | What methodological orientation was stated to underpin the study? e.g. grounded theory, discourse  analysis, ethnography,  phenomenology, content analysis | Our study employed a phenomenological descriptive approach utilizing in-depth interviews.  Data was inductively analysed. |
| 10. Sampling | How were participants selected? e.g. purposive, convenience, consecutive, snowball | Participants were purposively selected. |
| 11. Method of  approach | How were participants approached?  e.g. face-to-face, telephone, mail, email | Recruitment was conducted telephonically and through email by a senior researcher who is not involved in the day-to-day activities at the sites. |
| 12. Sample size | How many participants were in the study? | 34 healthcare providers participated in the study. |
| 13. Non-participation | How many people refused to participate or dropped out?  Reasons? | 36 participants were screened and invited to participate; all were eligible and 34 agreed to participate in the study. Data for all 34 participants were included in the final analysis.  Two participants declined participation, one did not respond to the invitation and one reported that she will not be available during data collection period. |
| Setting |  |  |
| 14. Setting of data  collection | Where was the data collected? e.g.  home, clinic, workplace | Interviews were held in private spaces within the healthcare facilities or nearby community-based organisations (CBOs). |
| 15. Presence of nonparticipants | Was anyone else present besides the participants and researchers? | There were no people present during the data collection besides participants and researchers. |
| 16. Description of  sample | What are the important  characteristics of the sample? e.g.  demographic data, date | Of the 34 participants, the majority (62%) were aged between 25-34 years. 88% were females and 56% were non-clinical staff. |
| Data collection | | |
| 17. Interview guide | Were questions, prompts, guides provided by the authors? Was it pilot tested? | Interviews were conducted using a semi-structured guide. The semi-structured interview guide was not piloted. |
| 18. Repeat interviews | Were repeat inter views carried out?  If yes, how many? | There were no repeat interviews. It was a single interview. |
| 19. Audio/visual  recording | Did the research use audio or visual recording to collect the data? | All interviews were audio recorded with the permission of participants. |
| 20. Field notes | Were field notes made during and/or after the interview or focus group? | Researchers did not make field notes. |
| 21. Duration | What was the duration of the interviews or focus group? | The interviews lasted 60 minutes on average. |
| 22. Data saturation | Was data saturation discussed? | We focused on capturing diverse perspectives, which resulted in a broad range of experiences rather than reaching data saturation on a single aspect. |
| 23. Transcripts  returned | Were transcripts returned to participants for comment and/or  correction? | Transcripts were not returned to participants for comment or correction. |
| Domain 3: Analysis and findings | | |
| Data analysis | | |
| 24. Number of data  coders | How many data coders coded the data? | Three researchers (FAC, FMM and SD) independently coded two transcripts using open coding, compared codes to ensure consistency and developed a preliminary coding framework. This was reviewed by the team, revised and the coding framework finalized, after which the remaining transcripts were analysed. |
| 25. Description of the  coding tree | Did authors provide a description of the coding tree? | There is no description of the coding tree. |
| 26. Derivation of  themes | Were themes identified in advance or derived from the data? | Themes were derived from the data in an inductive manner. |
| 27. Software | What software, if applicable, was used to manage the data? | Researchers used NVivo version 14 to code all  interviews. |
| 28. Participant checking | Did participants provide feedback on the findings? | Participants did not provide feedback on the findings. |
| Reporting | | |
| 29. Quotations  presented | Were participant quotations  presented to illustrate the themes/ findings? Was each quotation identified? E.g. participant number | Key findings of the study were reported with selected quotes in text. |
| 30. Data and findings consistent | Was there consistency between the data presented and the findings? | All findings were derived from the data and all themes are supported by illustrative quotes. |
| 31. Clarity of major themes | Were major themes clearly  presented in the findings? | Major themes are clearly defined by a paragraph title. |
| 32. Clarity of minor themes | Is there a description of diverse cases or discussion of minor  themes? | Findings of our study includes both major and minor themes that emerged on the data. During the analysis, we captured a range of participant perspectives to ensure understanding of provider’s perspectives on service integration in a setting where multiple PrEP methods are being introduced. |
